# Common Genetic Variants in miR-1206 (8q24.2) and miR-612 (11q13.3) Affect Biogenesis of Mature miRNA Forms

**DOI:** 10.1371/journal.pone.0047454

**Published:** 2012-10-15

**Authors:** Hye Kyung Kim, Ludmila Prokunina-Olsson, Stephen J. Chanock

**Affiliations:** Laboratory of Translational Genomics, Division of Cancer Epidemiology and Genetics, National Cancer Institute, Bethesda, Maryland, United States of America; The University of Arizona, United States of America

## Abstract

Cancer genome-wide association studies (GWAS) have identified many common genetic markers located in non-coding regions of the genome. Two notable examples are the multi-cancer susceptibility regions, 8q24.2 and 11q13.3. Since these GWAS signals localize to gene-poor regions, we investigated genetic variants within pre-microRNA (pre-miRNA) transcripts as a possible link between the GWAS findings and the associated molecular phenotypes. Across the two regions, which contain 37 miRNAs genes, we explored genetic variants by surveying public databases and conducting targeted resequencing. Specifically, we investigated one common single nucleotide polymorphism (SNP) within miR-1206 on 8q24.2 and two SNPs within miR-612 on 11q13.3. Though these variants are not correlated with known GWAS signals, we conjectured that they might be important for function of corresponding miRNAs. To test the functional significance of these genetic variants, we cloned both allelic forms of miR-1206 and miR-612 pre-miRNA into expression vectors and assessed biogenesis of mature miRNA-forms. The two SNPs within miR-612 significantly affected expression of mature miR-612 in a cell-type specific manner; enhancement in prostate cancer cell lines, reduction in colon cancer cells, and no effect in breast cancer cell lines. The SNP within miR-1206 also affected expression of mature miR-1206, but not in a cell-type specific manner. Future studies should identify targets of miR-1206 and miR-612 and help understand the biological roles of these miRNAs and their possible role in carcinogenesis.

## Introduction

Genome-wide association studies (GWAS) have identified over 220 cancer-associated genetic variations [1]. Many of the variants map to intergenic regions and of those that map to genes, only few have been shown to influence disease risk through coding variants. In this regard, it is possible that many GWAS-identified loci act through perturbations of regulation of genes or genomic elements, either locally or at a distance.

It is notable that two regions associated with multiple cancers on chromosomes 8q24.2 and 11q13.3 map to non-coding regions [2]–[12]. While the molecular mechanisms underlying the genetic associations in these regions have not been fully explained, a number of reports have suggested that non-coding variants influence regulatory elements, such as long-range enhancers of neighboring target genes, which could contribute to susceptibility to specific cancers [13], [14]. Recently, it was reported that the protective haplotype for the 11q13.3 renal signal shows reduced binding of HIF-2α and allelic imbalance in *CCND1* expression [15]. The common variant, rs6983267 on 8q24.2 is linked to regulation of the *MYC* proto-oncogene in colorectal cancer [16], [17]. Other studies have explored the possible functional effects of the long-range enhancer within 8q24.2 regions using a transgenic mouse model [18]. Alternatively, the non-coding variations could also affect expression or function of microRNAs (miRNA) in the associated regions either by affecting miRNA biogenesis or miRNA activity.

MicroRNA (miRNA) are small non-coding RNAs that control gene expression by post-transcriptional mechanisms [19]. Many miRNA genes can be found in the vicinity of chromosomal fragile sites and in genomic regions linked to cancer in either humans or mice, suggesting the possible role of miRNAs in genome instability [20]–[22]. miRNAs are initially transcribed as large precursor transcripts, which are subsequently processed into smaller (∼60 nt) hairpin shaped precursor miRNAs (pre-miRNA) by the Drosha/DGCR8 complex in the nucleus [23]. Upon export into the cytosol, enzymatic fragmentation by Dicer produces the mature functional ∼22 nt miRNA. Genetic variants may affect miRNA biogenesis, the processing from pre-miRNA to mature miRNA [24], [25]. Recent studies have suggested associations between specific miRNAs and a spectrum of chronic diseases, including cancer (e.g. cervical cancer, ovarian cancer, and lung cancer) [26]–[29]. Pilot studies have implicated genetic variants in miRNA or in their processing machinery genes in cancer risk [27], [30], [31].

In the present study, we investigated genetic variants located within pre-miRNA sequences in the two multi-cancer regions, 8q24.2 and 11q13.3. Each of these gene-poor regions harbors several independent signals, many of which are cancer-specific. We have investigated common SNPs, with minor allele frequencies (MAFs) more than 5%, by exploring public databases and performing targeted resequencing in SNP500 and HapMap samples. Functional consequences of three SNPs in miR-1206 (8q24.2) and miR-612 (11q13.3) were evaluated by *in vitro* studies. We uncovered distinct effects of pre-miRNA SNPs in mature miRNA biogenesis, whereby the same SNPs resulted in different levels of mature miRNA expression in a cell-type -specific manner. We expect that these results, together with future studies on miR-612 and miR-1206 target genes, could help clarify potential roles of miRNAs in these cancer risk loci.

## Results

### Cataloguing miRNA Genes in the Cancer Risk Loci 8q24.2 and 11q13.3

Both 8q24.2 and 11q13.3 loci are gene-poor regions known to harbor a number of miRNA genes. For a complete annotation, we searched for miRNA genes across the two regions (8q24, chr position 117,700,001–146,274,826 and 11q13, chr position 63,100,001–76,700,000) using miRBase database version 18 (Wellcome Trust Sanger Inst.). We identified 20 miRNA genes in 8q24 and 17 miRNA in 11q13 (**Table 1**).

**Table 1:** List of miRNAs in 8q24.2 and 11q13.3 catalogued in the miRNA database (v18).

Locus	miRNA	Chromosomeposition (hg18)	SNP
8q24	hsa-miR-1204	128877390–128877456	
8q24	hsa-miR-1205	129042061–129042123	
8q24	hsa-miR-1206	129090326–129090384	rs2114358
8q24	hsa-miR-1207	129130580–129130666	
8q24	hsa-miR-1208	129231555–129231574	rs56863230,rs2648841
8q24	hsa-miR-548d1	124429455–124429551	
8q24	hsa-miR-3686	130565485–130565570	rs6997249
8q24	hsa-miR-3673	130577270–130577371	
8q24	hsa-miR-3669	130578776–130578856	
8q24	hsa-miR-5194	131089762–131089881	rs78360334
8q24	hsa-miR-30b	135881945–135882032	
8q24	hsa-miR-30d	135886301–135886370	
8q24	hsa-miR-151a	141811845–141811934	
8q24	hsa-miR-1302-7	142865510–142865581	
8q24	hsa-miR-4472-1	143255607–143255686	rs28655823
8q24	hsa-miR-4664	144887241–144887311	rs6981062
8q24	hsa-miR-937	144967115–144967200	
8q24	hsa-miR-661	145091347–145091435	
8q24	hsa-miR-939	145590172–145590253	
8q24	hsa-miR-1234	145596286–145596367	
**Locus**	**miRNA**	**Chromosome** **position (hg18)**	**SNP**
11q13	hsa-miR-1237	63892650–63892751	
11q13	hsa-miR-192	64415185–64415294	
11q13	hsa-miR-194-2	64415403–64415487	
11q13	hsa-miR-612	64968505–64968604	rs550894, rs12803915
11q13	hsa-miR-4690	65160357–65160416	
11q13	hsa-miR-4489	65173239–65173300	
11q13	hsa-miR-3163	66458481–66458553	
11q13	hsa-miR-4691	67557940–67558024	
11q13	hsa-miR-3164	68607220–68607302	
11q13	hsa-miR-548k	69807709–69807824	
11q13	hsa-miR-3664	70396023–70396121	
11q13	hsa-miR-3165	71460922–71460996	
11q13	hsa-miR-139	72003755–72003822	
11q13	hsa-miR-4692	72172223–72172285	
11q13	hsa-miR-548al	73787930–73788026	rs10437738,rs515924
			rs60917039
11q13	hsa-miR-4696	74108961–74109030	
11q13	hsa-miR-326	74723784–74723878	rs72561778

### Re-sequencing Analysis of miRNA Genes in 8q24.2 and 11q13.3

**Figure 1:**
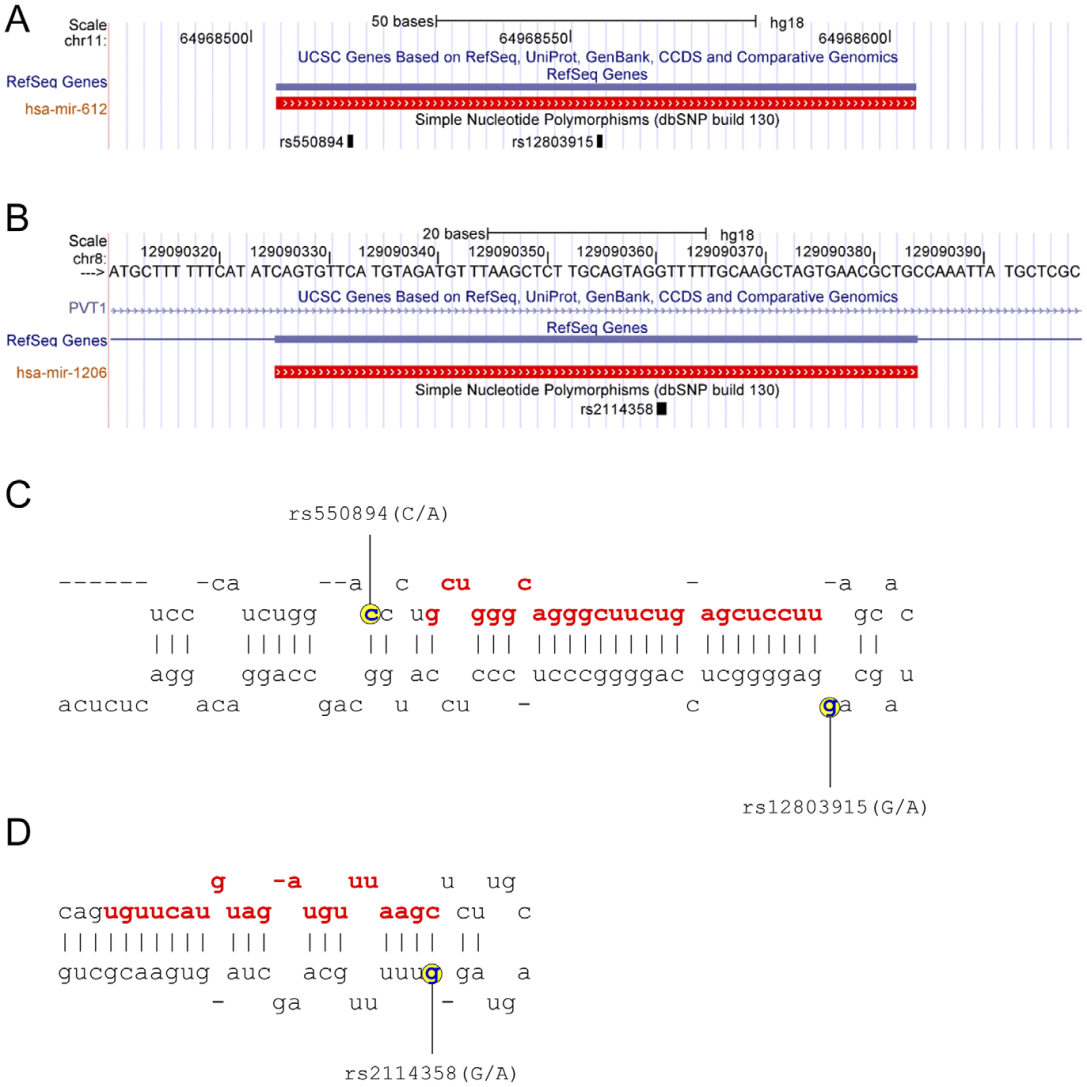
Genomic location and secondary structure of miR-612 and miR-1206. Annotation of miR-612 (**A**) and miR-1206 (**B**) using UCSC genome browser. Location of SNPs within pre-miR-612 (**C**) and pre-miR-1206 (**D**). Mature miRNA sequences are shown in red. SNP positions are indicated in yellow.

Screening for known genetic variants within miRNA genes in 8q24.2 and 11q13.3 regions using the dbSNP database (www.ncbi.nlm.nih.gov/projects/SNP/ *)* and 1000 genomes data (www.1000genomes.org) [32] revealed a small number of genetic variants, most of which had low MAFs in the public databases (data not shown). Thus, to address this issue in further detail, we re-sequenced the pre-miRNA regions of the twenty miRNA genes in 8q24.2 and of seventeen miRNA genes in 11q13.3 using 102 SNP500 cancer [33] samples (24 AFAM, 31 CAU, 23 HIS, 24 PARI) and of fourteen miRNA genes in 8q24.2 and of seven miRNA genes in 11q13.3 using 270 unrelated HapMap samples (90 CEU, 90 YRI, 45 CHB, and 45 JPT). As shown in **Table 2** **,** we were able to confirm previously reported SNPs and also identified new SNPs within pre-miRNA genes. Specifically, in 8q24.2, we identified one new SNP and confirmed seven known SNPs (rs2114358, rs56863230, rs2648841, rs6997249, rs78360334, rs28655823, and rs6981062). In 11q13, we confirmed six known (rs550894, rs12803915, rs10437738, rs515924, rs60917039, and rs72561778) and found one new SNPs (**Table 2**). Across the twenty miRNA genes in 8q24.2, seven contained genetic variants in pre-miR regions (miR-1206, miR-1208, miR-3686, miR-5194, miR-4772-1, miR-4664, and miR-939). Of seventeen miRNA genes in 11q13.3, only four contained SNPs (miR-612, miR-326, miR-548a1, and miR-1237) (**Table 2**). We found that one SNP in miR-1206 and two SNPs in miR-612 have MAFs greater than 5%. These variants were selected for our further study; rs2114358 in miR-1206 (8q24), as well as rs550894 and rs12803915 in miR-612 (11q13.3) (**Figure 1**). Analysis of the linkage disequilibrium (LD) pattern between rs2114358, rs550894, rs12803915 and cancer GWAS signals (**Supplementary** **Fig. 1**), showed that the miRNA variants were not correlated with the known 8q24.2 bladder, breast, colon and prostate cancer GWAS signals, namely rs16901979, rs6983267, rs4242382, and rs9642880, rs10088218 [8], [11], [34]–[36], or 11q13.3 prostate cancer GWAS signal, rs10896449 [36].

**Table 2:** SNPs in miRNA genes in 8q24.2 and 11q13.3 genotyped in SNP500 and HapMap samples.

miRNA	SNP	Allele^a^	CAU^b^	AFAM^c^	HIS^d^	PARI^e^	Hap CEU	Hap CHB+JPT	Hap YRI
**8q24.2**									
miR-939	novel^f^	C/T	0	0.02	0	0	0	0	0
miR-1206	rs2114358	G/A	0.41	0.22	0.26	0.45	0.42	0.34	0.22
miR-1208	rs56863230	G/C	0	0	0	0	0	0.6	0
miR-1208	rs2648841	G/T/A	0.97	0.67	0.83	0.38	0.94	0.56	0.84
	rs2648841	G/T/A	0.03	0.10	0.15	0.56	0.05	0.08	0.08
	rs2648841	G/T/A	0	0.23	0.02	0.06	0.01	0.08	0.08
miR-3686	rs6997249	G/A	0.27	0.1	0.2	0.15	N.A.	N.A.	N.A.
miR-4772-1	rs28655823	G/C	0.11	0.46	0.13	0.13	N.A.	N.A.	N.A.
miR-4664	rs6981062	A/G	0	0.04	0.02	0	N.A.	N.A.	N.A.
miR-5194	rs78360334	T/C	0	0	0	0.04	N.A.	N.A.	N.A.
**11q13.3**									
miR-326	rs72561778	A/G	0	0.02	0	0	0	0	0
miR-612	rs550894	C/A	0.11	0.10	0.37	0.27	0.06	0.25	0.1
miR-612	rs12803915	G/A	0.24	0.25	0.13	0.15	0.26	0.1	0.08
miR-1237	novel^g^	G/C	0	0.02	0	0	0	0	0
miR-548al	rs10437738	A/G	0	0.04	0.04	0	N.A.	N.A.	N.A.
	rs60917039	G/A	0	0.10	0	0	N.A.	N.A.	N.A.
	rs515924	A/G	0.15	0.1	0.1	0.27	N.A.	N.A.	N.A.

a allele 1/allele 2; allele frequencies are for underlined alleles. SNP500 samples:

b Caucasian (n = 31),

c African American (n = 24),

d Hispanic (n = 23),

e Pacific Rim (n = 24); HapMap samples: Hap CEU (n = 90), Hap CHB+JPT (n = 90), Hap YRI (n = 90).

f miR-939 novel SNP at chr position 145590173;

g miR-1237 novel SNP at chr position 63892701 (based on hg 18).

N.A. = not assessed.

### The Effects of pre-miRNA SNPs in miR-1206 and miR-612 on Generation of Mature miRNA in Human Cell Lines

**Figure 2:**
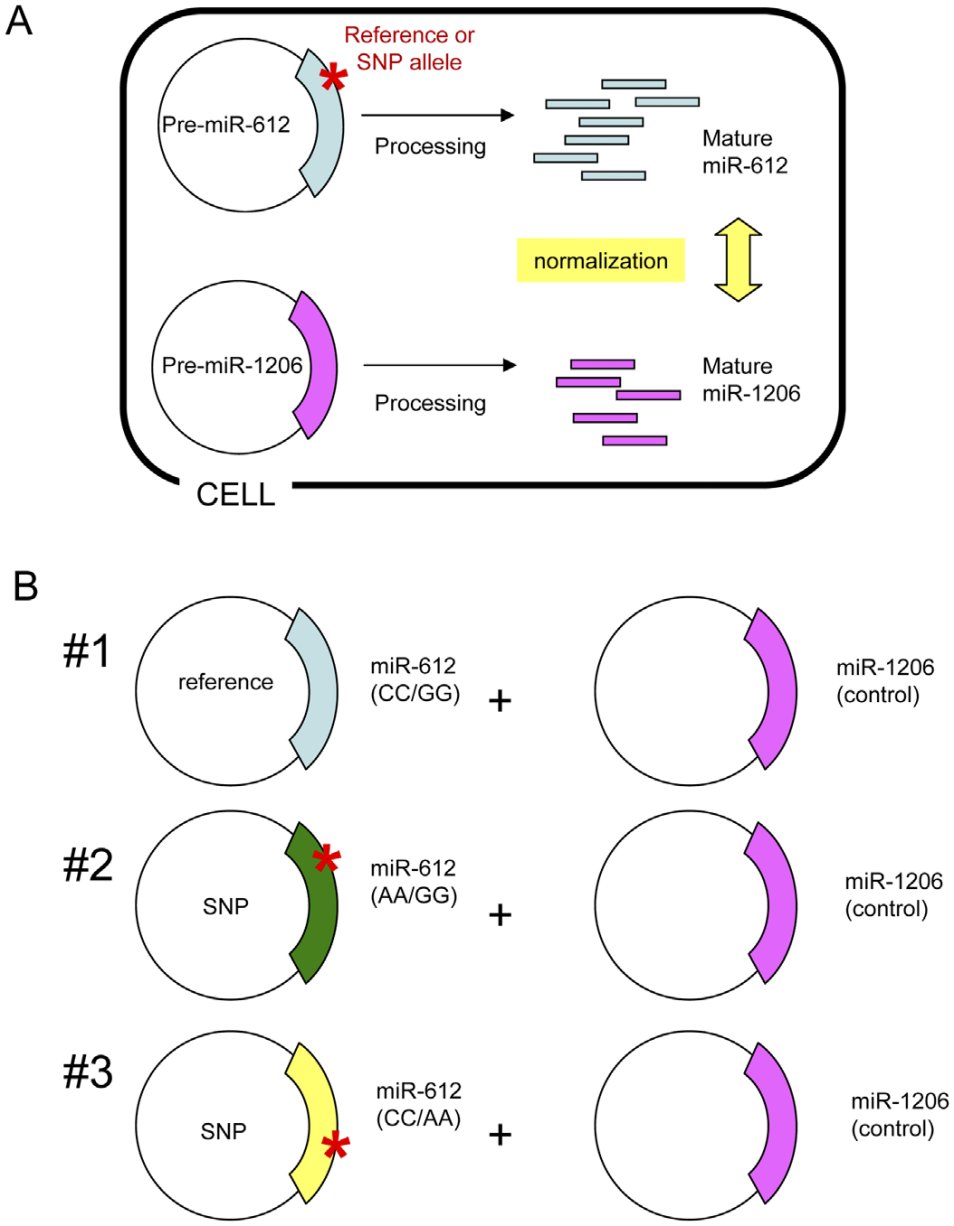
Schematic outline of experimental procedures. A. Co-transfection of allelic forms of miR-612 with a miR-1206 construct used as a normalization control. **B.** Co-transfection scheme for testing SNP effects on miR-612 processing. Three different combinations of allelic forms of miR-612 (#1∼#3) were co-transfected with control miR-1206 expression construct.

**Figure 3:**
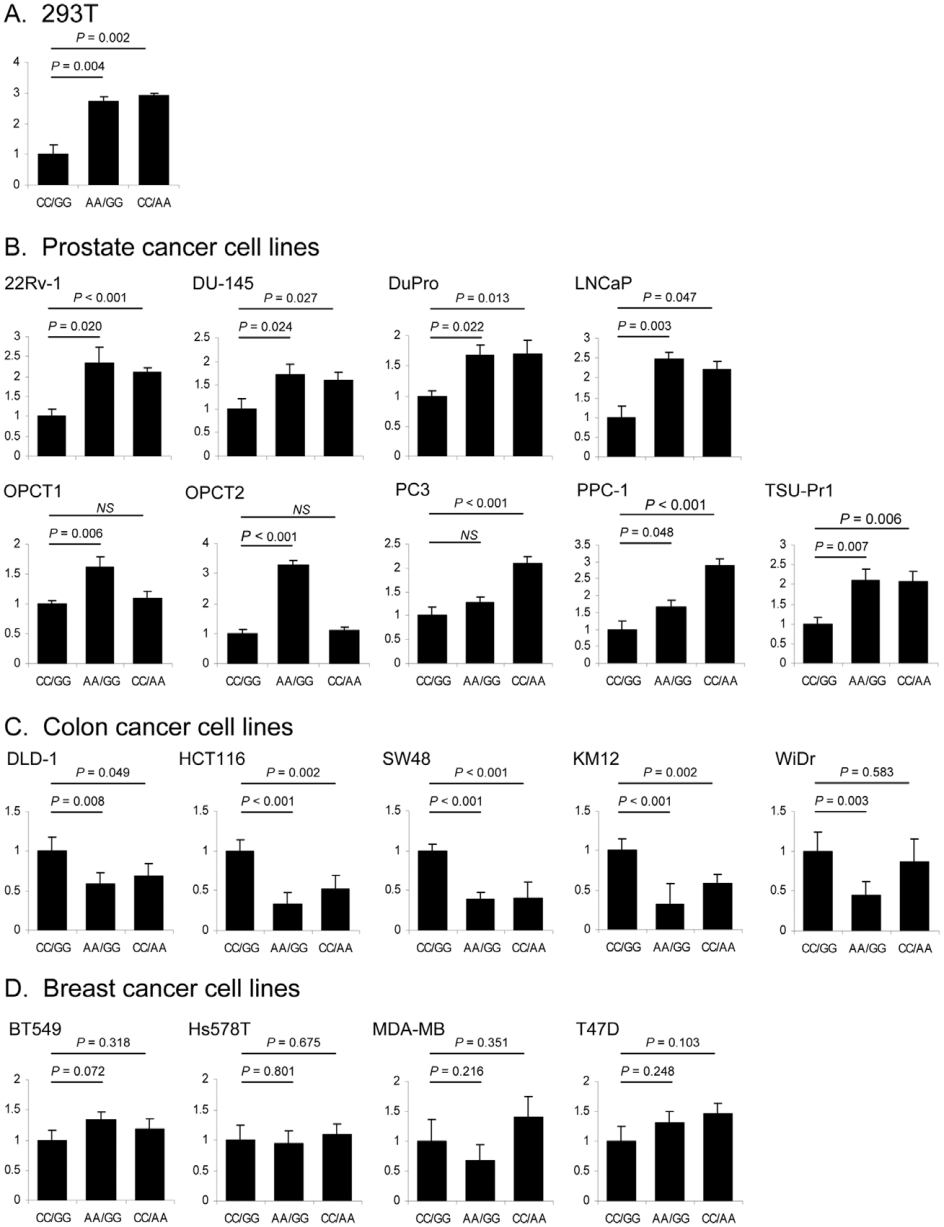
The effects of two SNPs in pre-miR-612 on expression of mature miR-612. A. Allelic forms of pre-miR-612 were cloned into miRNA expression vector pEGP-miR. Graphs show analysis of mature miR-612 expression in 293T cells, in relation to combination of rs550894 and rs12803915 genotypes: CC/GG (reference), AA/GG and CC/AA. **B.** In prostate cancer cell lines. **C.** In colon cancer cell lines. **D.** In breast cancer cell lines.

To assess whether pre-miRNA SNPs have functional consequences, we tested mature miRNA biogenesis of miR-612 and miR-1206 carrying alleles of the three selected SNPs (**Figure 2**). Allelic forms of pre-miRNAs were cloned into expression vectors and quantitative expression of mature miRNAs was evaluated by qRT-PCR with TaqMan miRNA assays after transient transfection into a panel of human cell lines (**Fig. 3** **and** **4**). Of note, mature miRNA was reverse transcribed using “stem-loop reverse transcription” primers, which are highly specific for the canonical mature miRNA sequence [37]. In fact, this method discriminates as little as one nucleotide differences in mature miRNAs and thus will detect only perfect matches to the mature miRNAs from miRBase database, thereby excluding from our detection allelic 3′-end miRNA. As an additional control ensuring the absence of potential interference of endogenous miRNA with transfected miRNA, we determined endogenous miRNA levels to establish a baseline level of expression, which was found to be negligible.

**Figure 4:**
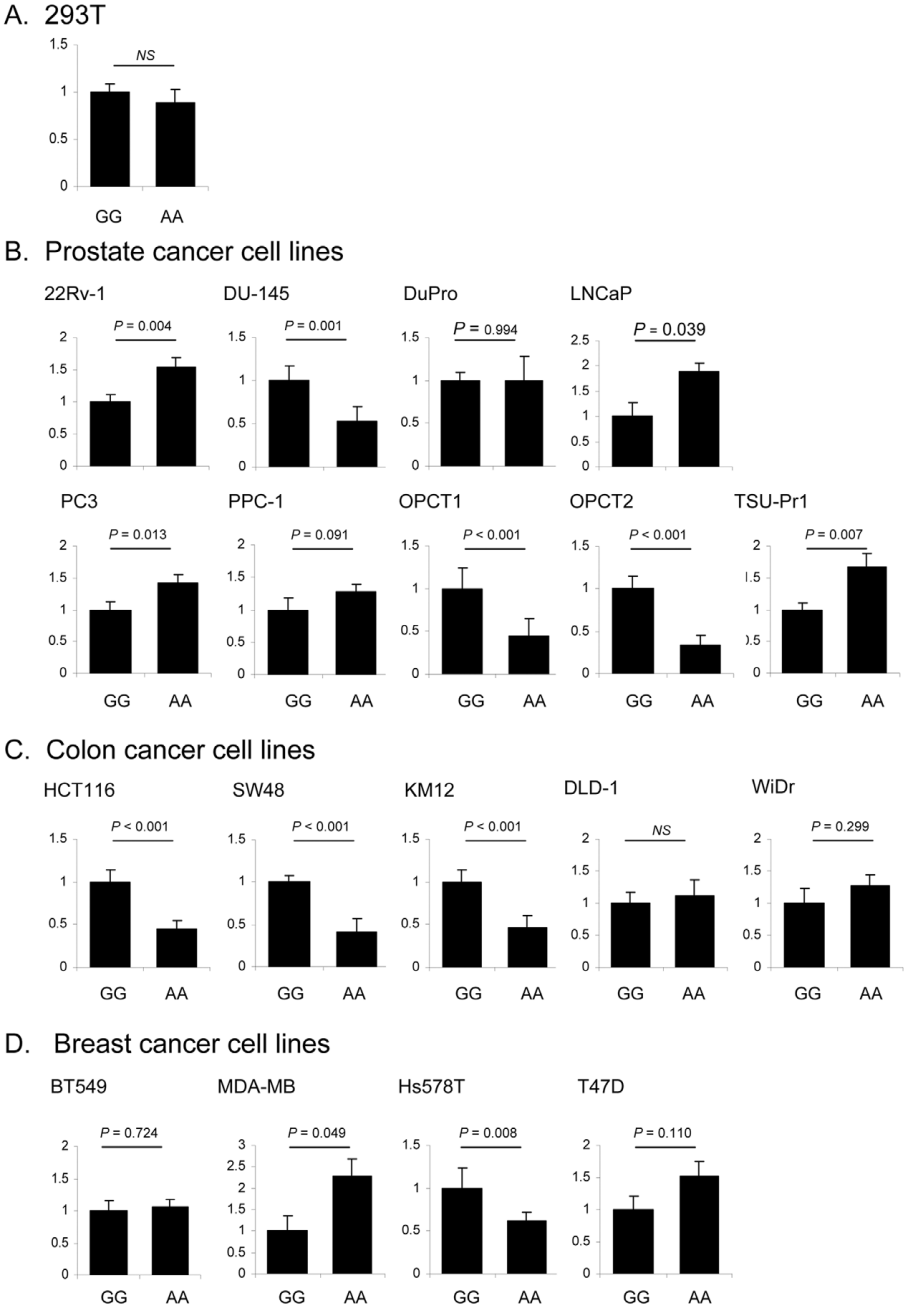
The effects of a SNP in pre-miR-1206 on expression of mature miR-1206. A. Allelic forms of pre-miR-1206 were cloned into miRNA expression vector pEGP-miR. Graphs show analysis of mature miR-1206 expression in 293T cells, in relation to rs2114358 GG and AA genotypes. **B.** In prostate cancer cell lines. **C.** In colon cancer cell lines. **D.** In breast cancer cell lines.

In 239T cells (normal embryonic kidney) we observed allelic effects of rs550894 and rs12803915 on expression of mature miR-612 (**Fig. 3A**). However, there was no allelic effect of rs2114358 on expression of mature miR-1206 (**Fig. 4A**). These results suggest that SNPs in pre-miRNA genes affect mature miRNA expression and that such functionality of SNPs can be effectively examined using this experimental system.

To examine whether the SNP alleles affect biogenesis of mature forms of miR-612 and miR-1206 in context of cancer cells, we tested a series of prostate cancer, colon cancer and breast cancer cell lines. In nearly all of the tested prostate cancer cell lines, we observed that the miR-612 rs550894 allele A conferred significant increase in mature miR-612 expression, with the only exception for PC3 cells, where the effect was not significant (**Fig. 3B**). Interestingly, we observed the same trend for the rs12803915 allele A, which significantly increased miR-612 expression in most of the prostate cancer cell lines, with the exception of OPCT1 and OPCT2 (**Fig. 3B**). In colon cancer cell lines DLD-1, HCT116, KM12, SW480 and WiDr, we found that rs550894 and rs12803915 alleles significantly decreased mature miR-612 expression (**Fig. 3C**). The allelic effects of pre-miRNA variations were absent in four breast cancer cell lines, BT549, HS578T, MDA-MB-468, and T47D (**Fig. 3D**). Together, these data suggest that SNP effects on pre-miRNA processing might be dependent on the cellular and tissue-specific context of the miRNA biogenesis machinery.

The effect of rs2114358 on miR-1206 expression in the same panel of human cell lines was diverse (**Fig. 4B**). While the majority of prostate cell lines, *i.e.* five out of nine, showed significant increase of mature miR-1206 expression, we also found that two cell lines, DuPro and PPC-1, did not show any effects and the effect in OPCT1 and OPCT2 was opposite, that is expression of mature miR-1206 was decreased. Among the four breast cancer cell lines tested, two cell lines did not display allelic effects, while mature miR-1206 expression was decreased in HS578T cells, and increased in MDA-MB-468 cells (**Fig. 4D**). In colon cancer cell lines, the effect was similar - mature miR-1206 expression was affected in some cell lines, while not changed in other cell lines (**Fig. 4C**).

Mature miRNAs are processed by the RNAase III ribonucleases Drosha and Dicer. A growing body of evidence indicates that there are variations in pre-miRNA processing efficiencies, resulting in miRNA length variants that differ from mature miRNA forms annotated in miRBase. These variations usually result in 1∼3 nucleotides longer transcripts at the 3′-ends of processed miRNAs [38], [39]. To examine whether the SNPs within pre-miR-612 and -1206 can affect processing of mature miRNAs with -3′-end length variants, next, we replicated our TaqMan miRNA expression results using an alternative strategy of miRNA quantification (**Supplementary** **Fig. 2**). The miScript reverse transcription method uses the poly(A) RNA polymerase to add a stretch of 3′ poly(A) ribonucleotides to the mature miRNA (Materials and Methods). cDNA is then synthesized from this RNA template using oligo-dT primers fused to oligonucleotide sequence that confers specificity to the RT-PCR. SYBR Green quantitative PCR with miRNA specific forward primers is then used to measure expression of mature miRNA. Using 22Rv-1 prostate cancer and HCT116 colon cancer cells, we showed that effects of SNPs on miRNA expression were replicated even for miRNAs with 3′-end variants (**Supplementary** **Fig. 2**). Thus we confirmed that expression of mature miRNAs, even with 3′-end variants is affected by pre-miRNA SNPs. Altogether, these results suggest allelic effects of these examined SNPs in individual cell lines, but these effects cannot be generalized as tissue-type specific.

**Figure 5:**
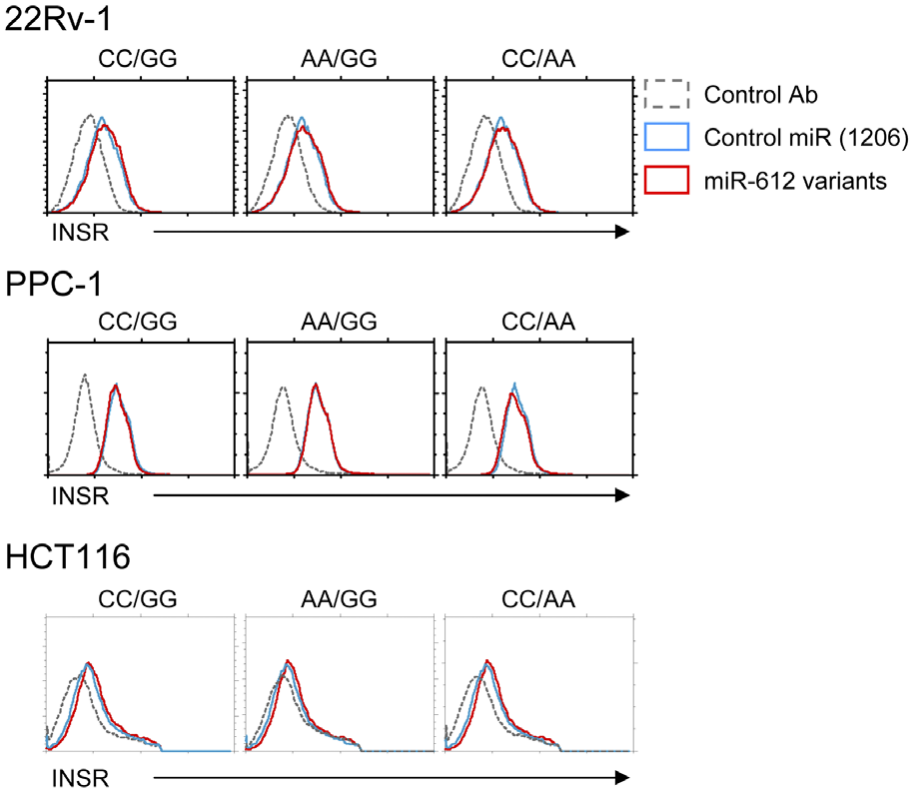
Analysis of surface expression of Insulin Receptor (INSR) in miR-612 transfected cancer cell lines. Cell surface insulin receptor (INSR) expression was determined by anti-INSR staining and flow cytometry24 hours post-transfection of the indicated miR-612 expression vectors. The results are representative of four independent experiments.

Finally, we wished to examine if such differential miRNA expression would show any biological effects. So far, no actual target gene has been identified for miR-612 or miR-1206, which makes analyzing the impact of differential miR-612 or miR-1206 expression levels quite difficult. Nevertheless, a single study reported a miR-612 target site at the 3′UTR of the insulin receptor (INSR), and proposed a potential association of insulin receptor expression/insulin signaling with colorectal cancer [40]. Since this was the only study that had an actual target gene reported for miR-612, we analyzed INSR expression upon miR-612 transfection. As shown in **Fig. 5**, INSR was found to be expressed on various cancer cell lines, though at different levels. Notably, transfection of allelic forms of pre-miR-612 did not result in any changes of INSR levels (**Fig. 5**). Either miR-612 does not strongly affect surface expression of INSR detectable with the antibody used in this experiment, or INSR is not a target of miR-612 in the cell lines and conditions tested. Analysis of differential effects of miR-612 expression still requires identification of a target gene of miR-612.

## Discussion

In the present study, we identified and characterized three common genetic variants in pre-miRNA genes within two cancer risk loci, 8q24.2 and 11q13.3. These gene-poor regions had been previously identified by cancer GWAS as associated with risk for multiple cancers, including cancers of the prostate, colon and breast [4], [6], [7], [9]–[12], [35], [41]. Notably, both 8q24.2 and 11q13.3 have also been reported as frequent chromosomal breaks in cancer, often a signature of genomic instability [20], [42]. We hypothesized that genetic variants within the miRNA genes might represent a possible link between GWAS findings and molecular phenotypes of these associations. Our search identified two miRNA genes, miR-1206 within 8q24.2 and its variant, rs2114358 and miR-612 within 11q13.3 and its two variants, rs550894 and rs12803915. We tested functional potential of these variants on biogenesis of mature forms of the corresponding miRNAs.

In general, we observed significant effects of these SNPs in individual cell lines, but these effects were cell line and cell type specific. There was a pronounced allelic effect of miR-612 in prostate and colon cancer cell lines, but not in breast. The allelic effect of miR-1206 was even more diverse, not allowing generalization across the cell lines. These data confirmed that select genetic variants can affect miRNA expression *in vitro*, but interpretation of these findings should be based on consideration of cell-type specificity.

Assessment of miRNA SNPs, however, indicate that the variants within miR-1206 and miR-612 are not strongly correlated with the published GWAS signals in 11q13.3 and 8q24.2 for breast, colon and prostate cancer as well as the bladder cancer GWAS signal [6], and the ovarian cancer susceptibility locus, >700 kb telomeric to *MYC* [41]. These results suggest that the miRNA SNPs tested in this study do not explain the GWAS signals in these regions.

Mutations or aberrant expression of genes in the miRNA biogenesis pathway have been documented to cause defective miRNA expression [43]. Another major pathway of impaired miRNA expression may involve SNPs in the miRNA genes [27], [44], [45]. miRNA genes are highly conserved, and any variants within these sequences as well as miRNA binding sites within target genes are considered to be under extreme negative selective pressure [46], [47]. There is preliminary evidence that such variation could be associated with increased risk for cancer but larger studies are needed to confirm these observations. [27] In this regard, identification of functional variants in miR-612 and miR-1206 genes may be an important advance in understanding the role of miRNA in general, regulation of the miRNA biogenesis and expression by genetic variants, specifically.

The 8q24 miR-1206 is of particular interest as it is part of long non-coding RNA (lncRNA) transcript of the *PVT1* gene. Recently, *PVT1* expression has been linked to SNP, rs378854 [48], which is within the same haplotype block and in complete LD with rs620861, a prominent GWAS signal for prostate cancer [10], [49]. Chromatin confirmation capture experiments further showed that DNA regions surrounding rs378854 physically interacted with the *PVT1* promoter, and suggested that predisposition to prostate cancer at 8q24.2 risk locus could be associated with *PVT1* expression. *PVT1* is a non-protein coding locus that harbors a number of non-coding RNAs and miRNAs. Specifically, miR-1206 is one of six annotated miRNAs found in the non-coding *PVT1* locus that also includes miR-1204, miR-1205, miR-1207-5P, miR-1207-3p and miR-1208. While overexpression of *PVT1* transcript has been documented in a variety of tumor tissues including breast and ovarian cancer and also in Hodgkin lymphoma [50], miR-1206 expression has been found in increased levels in B cell tumors such as Namalwa and CA-46 [51]. Interestingly, primary *PVT1* transcripts are significantly expressed in prostate tissues but mature miR-1206 expression was found to be undetectable in primary normal and tumor prostate samples [48]. A possible link between cancer and *PVT1* locus has been reported by identifying a role of p53 in *PVT1* expression. Accordingly, treatment of the DNA-damaging agent daunorubicin led to increased *PVT1* transcripts and to significantly increased miR-1206 expression in hepatocellular carcinoma SK-HEP1 cells, colon carcinoma PKO, and HCT116 cells in a p53-dependent manner [52]. The identities of miR-1206 target genes and their potential role in carcinogenesis, however, will require additional interrogation.

Dysregulation of miR-612 is also potentially a mechanism of increased cancer risk. miRNA profiling of colorectal cancer (CRC) tissue samples revealed a strong (>5 fold) increase in miR-612 expression in CRC samples over normal tissues [53]. In a case-control association study of sporadic colorectal cancer, a genetic variation in the 3′UTR of insulin receptor (INSR) expression, corresponding to a miR-612 target site, was significantly associated with increased cancer risk [40]. However, we could not validate INSR as a target of miR-612 in cell lines and conditions used in our study. Thus, the direct target genes of miR-612 still need to be identified and validated.

In conclusion, we provide data on allelic differences in the biogenesis of mature forms of miR-612 and miR-1206 in human cell lines. Still, it is necessary to explore expression patterns of miR-1206 and miR-612 in human normal and tumor tissues in relation to these genetic variants. Expression profiling and target gene identification of these miRNAs are needed to elucidate these issues in follow-up studies.

## Materials and Methods

### Resequencing of HapMap Samples

PCR primers for amplification of the pre-miRNA genomic regions were designed using Primer3 (http://frodo.wi.mit.edu/primer3/input.htm), based on the human genome reference sequence version hg18 (**Suppl.** **Table 1**). PCR products were confirmed by agarose gel electrophoresis, purified using Agencourt AMPure XP (Beckman Coulter Genomics, Danvers, MA) and sequenced with the BigDye Terminator Cycle Sequencing kit (Applied Biosystems, Foster city, CA) on an ABI Prism 3730x/DNA Analyzer (Applied Biosystems). Sequence analysis and SNP scoring was done with Sequencher 5.0 (Gene Codes Corp., Ann Arbor, MI) and Variant Reporter v1.0 (Applied Biosystems).

### Generation of miRNA Expression Constructs

The genomic regions corresponding to allelic variants of miR-1206 and miR-612 pre-miRNAs were PCR-amplified from HapMap DNA samples (with primers shown in **Suppl.** **Table 2**) and cloned into *Nhe*I and *Bam*HI sites of pEGP-miR vector (Cell Biolabs Inc). The constructs were validated by Sanger sequencing.

### Cell Lines and Transfection

All cell lines were obtained from ATCC (Manassas, VA) and the National Cancer Institute Developmental Therapeutics Program (NCI60 cell line panel) except when indicated otherwise. OPCT1 and OPCT2 cell lines were purchased from Asterand (Detroit, MI). DuPro and TSU-Pr1 and PPC-1 were kind gifts from Dr. M. Scott Lucia (Colorado Molecular Correlates Laboratory). Prostate cancer cell lines were 22Rv-1, DU-145, DuPro, LNCaP, OPCT1, OPCT2, PC3, PPC-1, and TSU-Pr1. Colon cancer cell lines were DLD-1, HCT116, KM12, SW480, and WiDr. Breast cancer cell lines were BT549, Hs578T, MDA-MB-468, and T47D. Since every cell line displayed different transfection efficiencies, each cell line was individually tested and optimized for using one of several established methods. MiRNA expression vectors were transfected using the following reagents and protocols, respectively: Lipofectamine 2000 (Invitrogen): PPC-1, 22Rv1, DuPro, TSU-Pr1, DLD-1, 293-T, HCT-116, SW480, KM12, and HS578T; Lipofectamine LTX (Invitrogen): BT-549, WiDr, PC3, and MDA-MB-468; 4D nucleofector (Lonza): LNCaP, Du145, OPCT1, OPCT2, LNCaP, and T47D.

All transfections were performed at least two separate times with biological triplicates for each of constructs and controls. The pre-miRNA expression vectors co-express GFP, which allowed us to determine the transfection efficiency by calculating the percentage of GFP+ cells using fluorescent microscopy (Nexcelom Bioscience). Only transfections with greater than 50% efficiency were used for RNA extraction and further analysis.

### miRNA Extraction and TaqMan Assays for Mature miRNA Quantification

Total RNA was extracted from transfected cells using the *mir*Vana miRNA isolation kit (Ambion). To evaluate expression of mature miRNAs, we used stem–loop real-time quantitative reverse transcriptase PCR (qRT-PCR) TaqMan miRNA assays (TM001579 and TM002878 for miR-1206 and miR-612, respectively, ABI). We co-transfected miR-1206 and miR-612 constructs and measured expression of both transcripts in all samples. For example, miR-612 expression was used to normalize expression of miR-1206, and miR-1206 was used for normalization of miR-612 expression. Expression analysis in samples transfected with single miRNA constructs was used as positive and negative controls. Fold difference for expression of allelic forms of miRNA constructs was calculated using the equation 2^ΔΔct^, where ΔCt = Ct(target miRNA) – Ct(control miRNA) and ΔΔCt = ΔCt allele 1 - ΔCt allele 2. *P*-value <0.05 was considered to be statistically significant. The graphs were generated with MS-Excel and included the summary of two independent experiments in biological triplicates. Bar graphs show mean +/− SEM.

### miScript II Reverse Transcription and miScript SYBR Green Quantitative PCR Assays for Analysis of Expression of Mature miRNAs

To validate TaqMan miRNA assay results, expression of mature miRNAs was analyzed with an alternative method using the poly(A) RNA polymerase based miScript II reverse transcription system (QIAGEN). This method has the advantage of detection of all potential 3′-end variations in mature miRNA. Total RNA was isolated using miRNeasy (QIAGEN) and reverse transcribed into cDNA using miScriptII. Mature miRNA was detected using the “miScript universal primer” and the miRNA-specific miScript primer assay (both from QIAGEN). Control co-transfections, signal normaliztaion and data analysis were performed the same way as described above for TaqMan miRNA assays.

### Flow Cytometry

Single cell suspensions of transfected cell lines were analyzed on a FACSCalibur flow cytometer (Becton Dickinson). Dead cells were excluded by forward and side scatter gating. Phycoerythrin -conjugated anti-human INSR (CD220) antibodies were purchased from Biolegend. Data were analyzed by CellQuest (Becton Dickinson) and FlowJo (TreeStar Inc.).

### Statistical Analysis

Data were evaluated using the two-sided unpaired Student’s *t*-test.

## Supporting Information

Figure S1 **Linkage Disequilibrium (LD) plots of 8q24.2 and 11q13.2 regions.** The plots are based on in HapMap (CEU) samples and include genetic variants within miR-1206 and miR-612 genes and GWAS signals. Numbers in blocks indicate *r* ^2^ values. **A.** LD plot of two miR-612 SNPs, rs550894 and rs12803915, and the 11q13 GWAS signal rs10896449. **B.** LD plot of miR-1206 SNP rs2114358 and the 8q24.2 GWAS signals rs16901979, rs6983267, rs4242382, rs9642880, and rs10088218.
(PDF)

Figure S2 **Effects of 2 SNP in pre-miR-612 and pre-miR-1206 on expression of mature miRNAs.** Reference and allelic forms of pre-miRs were expressed in 22Rv-1 and HCT116 cells. Expression of mature miRNA was determined using the poly(A) RNA polymerase based miScript reverse transcription system (QIAGEN) followed by specific miScript SYBR Green quantitative PCR assays. A. expression of mature miR-612 in relation to combination of rs550894 and rs12803915 genotypes: CC/GG (reference), AA/GG and CC/AA. B. Expression of mature miR-1206 expression in relation to rs2114358 GG and AA genotypes.
(PDF)

Table S1 **Oligonucleotide sequences and their corresponding chromosomal positions for amplifying pre-miRNA genomic regions (based on hgv18).**
(DOC)

Table S2 **Oligonucleotide sequences used to amplify pre-miR-1206 and pre-miR-612 regions for expression vector cloning.**
(DOC)
