# Supplementary material for: Common Genetic Variants in miR-1206 (8q24.2) and miR-612 (11q13.3) Affect Biogenesis of Mature miRNA Forms
Source: PLoS One. 2012 Oct 15;7(10):e47454. doi: 10.1371/journal.pone.0047454 (PMC3471815; doi:10.1371/journal.pone.0047454)
Supplement: Figure S1 — Linkage Disequilibrium (LD) plots of 8q24.2 and 11q13.2 regions. The plots are based on in HapMap (CEU) samples and include genetic variants within miR-1206 and miR-612 genes and GWAS signals. Numbers in blocks indicate r 2 values. A. LD plot of two miR-612 SNPs, rs550894 and rs12803915, and the 11q13 GWAS signal rs10896449. B. LD plot of miR-1206 SNP rs2114358 and the 8q24.2 GWAS signals rs16901979, rs6983267, rs4242382, rs9642880, and rs10088218. (PDF) [file pone.0047454.s001.pdf]

Supplementary Figure 1

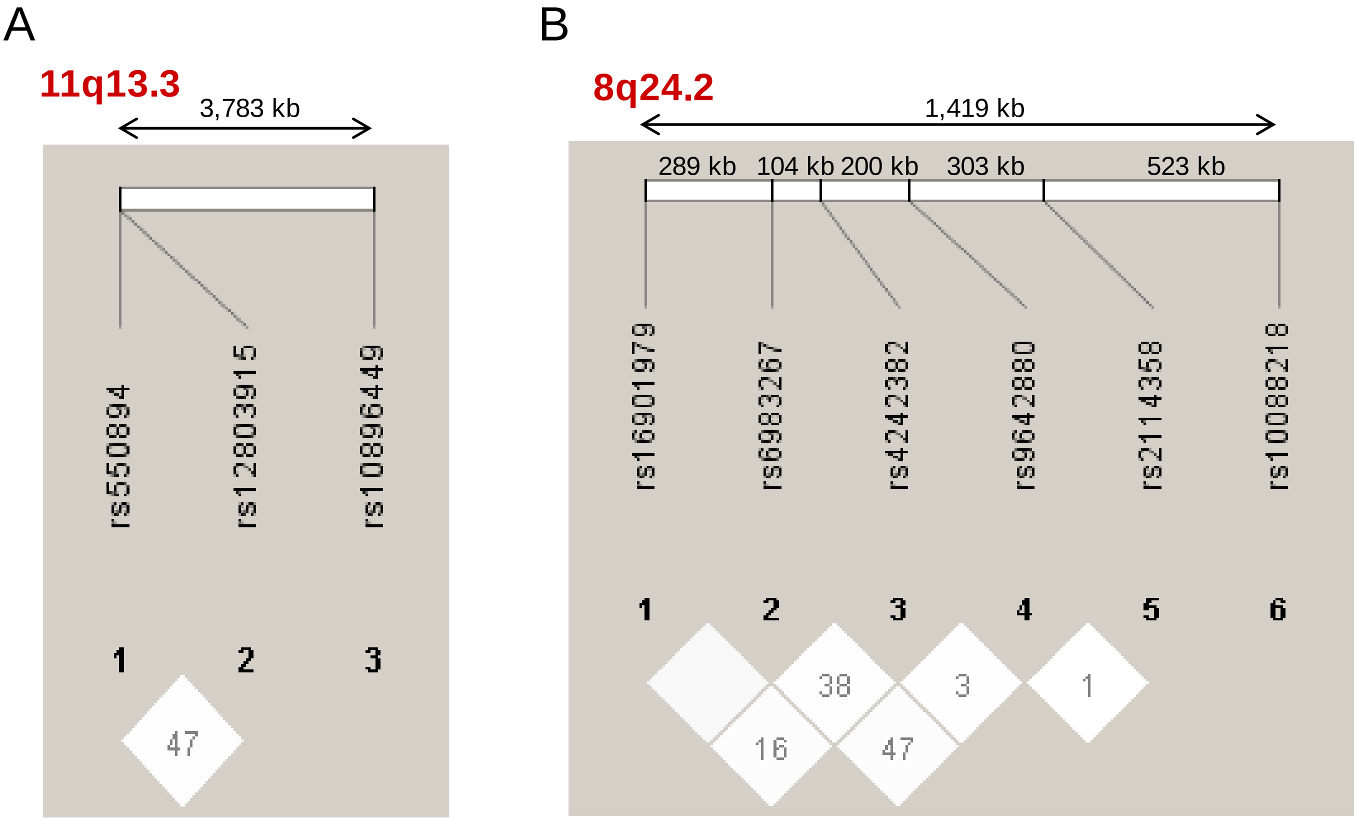

Supplementary Figure 1. Linkage Disequilibrium (LD) plots of 8q24.2 and 11q13.2 regions. The plots are based on in HapMap (CEU) samples and include genetic variants within miR-1206 and miR-612 genes and GWAS signals. Numbers in blocks indicate r^2 values.

A. LD plot of two miR-612 SNPs, rs550894 and rs12803915, and the 11q13 GWAS signal rs10896449.

B. LD plot of miR-1206 SNP rs2114358 and the 8q24.2 GWAS signals rs16901979, rs6983267, rs4242382, rs9642880, and rs10088218.
