# Supplementary material for: Common Genetic Variants in miR-1206 (8q24.2) and miR-612 (11q13.3) Affect Biogenesis of Mature miRNA Forms
Source: PLoS One. 2012 Oct 15;7(10):e47454. doi: 10.1371/journal.pone.0047454 (PMC3471815; doi:10.1371/journal.pone.0047454)
Supplement: Figure S2 — Effects of 2 SNP in pre-miR-612 and pre-miR-1206 on expression of mature miRNAs. Reference and allelic forms of pre-miRs were expressed in 22Rv-1 and HCT116 cells. Expression of mature miRNA was determined using the poly(A) RNA polymerase based miScript reverse transcription system (QIAGEN) followed by specific miScript SYBR Green quantitative PCR assays. A. expression of mature miR-612 in relation to combination of rs550894 and rs12803915 genotypes: CC/GG (reference), AA/GG and CC/AA. B. Expression of mature miR-1206 expression in relation to rs2114358 GG and AA genotypes. (PDF) [file pone.0047454.s002.pdf]

Supplementary Figure 2

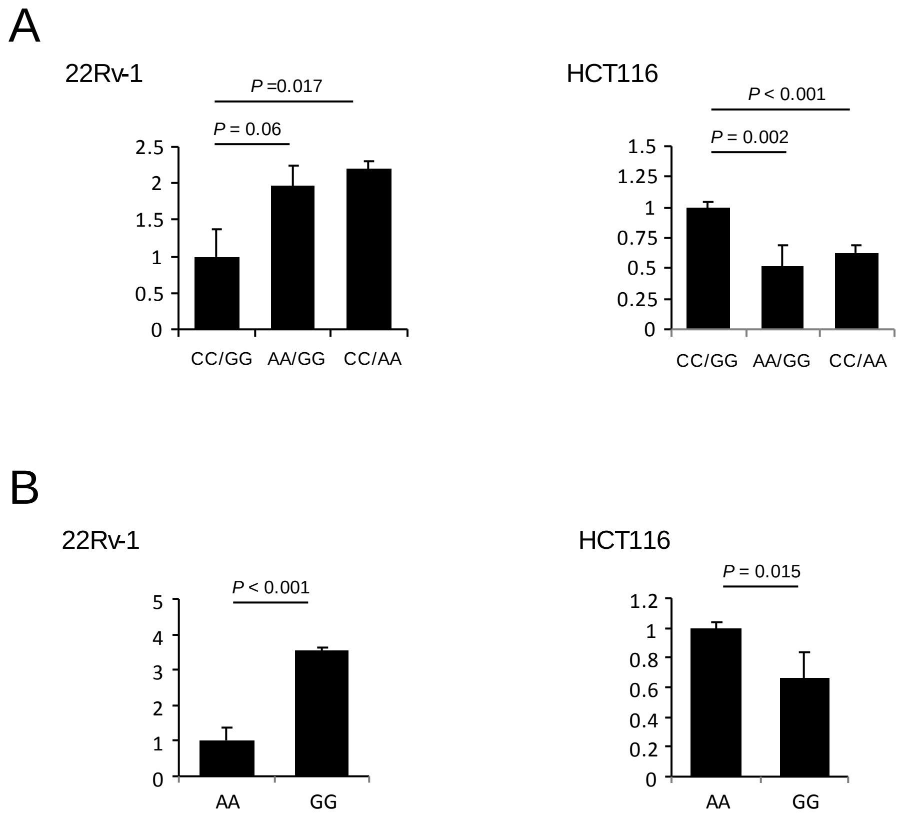

Supplementary Figure 2. Effects of 2 SNP in pre-miR-612 and pre-miR-1206 on expression of mature miRNAs.

Reference and allelic forms of pre-miRs were expressed in 22Rv-1 and HCT116 cells. Expression of mature miRNA was determined using the poly(A) RNA polymerase based miScript reverse transcription system (QIAGEN) followed by specific miScript SYBR Green quantitative PCR assays. **A.** expression of mature miR-612 in relation to combination of rs550894 and rs12803915 genotypes: CC/GG (reference), AA/GG and CC/AA. **B.** Expression of mature miR-1206 expression in relation to rs2114358 GG and AA genotypes.
