# Supplementary material for: Common Genetic Variants in miR-1206 (8q24.2) and miR-612 (11q13.3) Affect Biogenesis of Mature miRNA Forms
Source: PLoS One. 2012 Oct 15;7(10):e47454. doi: 10.1371/journal.pone.0047454 (PMC3471815; doi:10.1371/journal.pone.0047454)
Supplement: Table S1 — Oligonucleotide sequences and their corresponding chromosomal positions for amplifying pre-miRNA genomic regions (based on hgv18). (DOC) [file pone.0047454.s003.doc]

**Suppl.Table 1**

Oligonucleotide sequences and their corresponding chromosomal positions for amplifying pre-miRNA genomic regions (based on hgv18)

| **Chromosome position** | **Primer** |
| --- | --- |
| chr8:128877135-128877643 | 5’-CAGGGACTGTGTCTGCTGTG-3’  5’-GAAGCCGTTTATGCAAAAATTC-3’ |
| chr8:129041828-129042369 | 5’-GACAACAGCTTGGGGACATT-3’  5’-CCCCAAACACACACACAAGA-3’ |
| chr8:129090046-129090559 | 5’-TGGGTTCCTCTGCTTCTCTC-3’  5’-GCAGATTTCTAATACTTTATCTGCTTT-3’ |
| chr8:129130378+129130883 | 5’-GCCCAGGGTTTTCAGCTT-3’  5’-CAGAATCCCCAGACCTTGAC-3’ |
| chr8:129231466+129231987 | 5’-CCTTTGGCCTCAAATGCTAA-3’  5’-TGGTTTTCATCTGGTCTGCAT-3’ |
| chr8:124429161-124429673 | 5’-ATTCACACCAGGGATTGGAA-3’  5’-GCTCTCCAGCCATTTCTTCA-3’ |
| chr8:130565332-130565839 | 5’-GTTTCATCAAATGGCCCAAG-3’  5’-CCCCACCCTCATCTCATAAA-3’ |
| chr8:131089661-131090169 | 5’-CTTTCTGCCAGGTCCATAGC-3’  5’-CAAACAGGCTTGCAAATGAA-3’ |
| chr8:135881742-135882265 | 5’-GGCATAGTAGCGTGCCTGTA-3’  5’-TGCAACCATGCTTTCTTCTG-3’ |
| chr8:135886031-135886542 | 5’-TGCACTTGGTGAACCACTTT-3’  5’-ATTGACAGGGCAGAAACAGG-3’ |
| chr8:141811594-141812107 | 5’-TGACTAGCCTTCACCCTCTGA-3’  5’-TGAATGCTCAACTACCTGTGCT-3’ |
| chr8:142865264-142865798 | 5’-GCTGGTACCTCGTGGCTAGA-3’  5’-GTAGAGGAGAGCTGGGAGGA-3’ |
| chr8:143255454-143255980 | 5’-AGCGTTCTATTTCGGCTCAC-3’  5’-TTTATGGGTGTGCAGGGAGT-3’ |
| chr8:144887102-144887531 | 5’-AGGAAGTGGGGTTCCTGAGT-3’  5’-CTAGCTGGAGTCTGGGTGCT-3’ |
| chr8:144966961-144967476 | 5’-AAGTGCTGGTGCGTGGAG-3’  5’-CCAGGCACACTAACCGATG-3’ |
| chr8:145091086-145091598 | 5’-TTGGGATCTGATCTGCATAGG-3’  5’-CAGTCAGGTCTGGCTGGA-3’ |
| chr8:145589944-145590464 | 5’-ATGAAGAGCATTTCGCTGCT-3’  5’-TTTTTGCTGAGCCCTTCAGT-3’ |
| chr8:145596004-145596540 | 5’-GTGGATAGGGGAGTGTGTGG-3’  5’-GCCTTGTCAAAGTGGAACG-3’ |
| chr11:63892510+63893010 | 5’-CTCCAGGACTCGCCCTTCT-3’  5’-GCCCTACTCCCCTCAAATTC-3’ |
| chr11:64414977-64415500 | 5’-CCCGGTCTCCAATTGGTTC-3’  5’-AAGAGTATTGCAGCCCAAGG-3’ |
| chr11:64415131-64415674 | 5’-GACTTTGCCCAGGAAGGAGT-3’  5’-CTCTGCTGACTGCTGGACAC-3’ |
| chr11:64968290+64968825 | 5’-CCCACCTGGTAGTCCTCAGA-3’  5’-CCAGCCTTTCTCCTGTCACT-3’ |
| chr11:65160182-65160659 | 5’-GTGTCCCTCCTGATCCCTTT-3’  5’-CCAGGCTCCACTTTCCACT-3’ |
| chr11:65173062-65173551 | 5’-ATCCCCATTTCACAGATGGA-3’  5’-AGAGGGAAGGCCAAGAGTGT-3’ |
| chr11: 66458316-66458773 | 5’-CCCATTAACAGAGCCACCAT-3’  5’-CCAGGAGGTAGAGGTTGCAG-3’ |
| chr11: 67557809-67558294 | 5’-CTCTGGTCAGACCCCTGTGT-3’  5’-GCTGGGAAAACCTTCAAATG-3’ |
| chr11: 68607195-68607599 | 5’-AAGGCCTCAGTGCTATCCAC-3’  5’-AGCTACTCTGTGGCCTGGAG-3’ |
| chr11:69807564-69808137 | 5’-TGTTTGGTGGTGTTGAGAGG-3’  5’-ATGGCAAATTCAGTGTGTGC-3’ |
| chr11:70395809-70396317 | 5’-GTAGGATGATGCGTGTGTGG-3’  5’-GGGTCTCTCTTCCCTCCATC-3’ |
| chr11:71460818-71461202 | 5’-GCAGGCAATTACCTCACTCC-3’  5’-TTGAAGGGCCAGTTTCTTTG-3’ |
| chr11:72003545-72004058 | 5’-TCTTCCCATTCCTTCATCCA-3’  5’-GGAGACTAGTGGTGGGGAGA-3’ |
| chr11:72172065-72172547 | 5’-CCCATCCCTTCTAAACCACA-3’  5’-AGCTCTCTAGGGTGGGGAAG-3’ |
| chr11:73787782-73788281 | 5’-AGGTACAGGGGCGTAGTCCT-3’  5’-TCTCAGCATTTCCCAGACCT-3’ |
| chr11:74108689-74109188 | 5’-CCATTGCCTGAGCTCTTTTC-3’  5’-GGCCCTGGAATTGTGTAAAA-3’ |
| chr11:74723593-74724059 | 5’-AGCAATCCTCCCGTCTCAG-3’  5’-GGCGCCTAAAGCTTACAGAG-3’ |
