# Supplementary material for: Common Genetic Variants in miR-1206 (8q24.2) and miR-612 (11q13.3) Affect Biogenesis of Mature miRNA Forms
Source: PLoS One. 2012 Oct 15;7(10):e47454. doi: 10.1371/journal.pone.0047454 (PMC3471815; doi:10.1371/journal.pone.0047454)
Supplement: Table S2 — Oligonucleotide sequences used to amplify pre-miR-1206 and pre-miR-612 regions for expression vector cloning. (DOC) [file pone.0047454.s004.doc]

**Suppl. Table 2**

Oligonucleotide sequences used to amplify pre-miR-1206 and pre-miR-612 regions for expression vector cloning

miRNA Chr. Position Primer sequence

_____________________________________________________________________________________

miR-1206 chr8:129090202+129090485 5’-TCTTTATTCTGCCCACTCAGC-3’

(rs2114358) 5’-TGTCACTTGATAAAACAGTGATAGG-3’

miR-612 chr11:64968428+64968688 5’-GTAGAAGGCACCCAGCTCTC-3’

(rs550894) 5’-ACCACTGCTGTTGGCTCAG-3’

(rs12803915) _____________________________________________________________________________________
